# Editorial: New insights in *Mycobacterium tuberculosis*

**DOI:** 10.3389/fcimb.2022.1020267

**Published:** 2022-10-13

**Authors:** Kalimuthusamy Natarajaseenivasan

**Affiliations:** ^1^ Department of Microbiology, Bharathidasan University, Tiruchirappalli, India; ^2^ Department of Neural Sciences, Lewis Katz School of Medicine, Temple University, Philadelphia, PA, United States

**Keywords:** *Mycobacterium tuberculosis*, risk factors, diagnosis, biomarkers, therapy

Tuberculosis (TB), resulting from infection by the bacterium *Mycobacterium tuberculosis (Mtb)*, is one of the top 10 causes of death worldwide as per the World Health Organization (WHO). Approximately one-third of the World’s population is latently infected with *Mtb*, and this has a dramatic impact on the quality of life of the patients. To improve the battle against *Mtb*, we first need to understand the host-pathogen interactions. Secondly, developing new diagnostic tools or improving the sensitivity of the one already existing will help diagnose the disease in patients effectively, this will allow early treatment of the infected persons to avoid the late stage of the disease and its spread to others. This should be followed by developing some improvised therapeutic approaches.

Our Research Topic entitled “*New Insights in Mycobacterium tuberculosis*” emphasized the submission of original research papers describing novel approaches to understanding molecular pathogenesis, improvised diagnosis, and therapeutics with clinical trials. The overwhelming response from the authors indeed excited us and resulted in 10 articles with more than 12,000 views so far. These interesting novel works will benefit the readers and society.

Our collection includes exciting work about relapse, re-infection, and the current situation of recurrence of TB in Jiangsu, China (Shao et al.). In this work, they adapted population-based surveillance on culture-positive TB cases and systematically implemented MIRU-VNTR for drug resistance and genotype detection. The outcome of the study revealed that relapse and re-infection contributed equally to the situation of recurrence of TB in Jiangsu, China.

Ning et al. practiced the subunit vaccine ESAT-6:c-di-AMP through the intranasal route, which elicited a significant immune response to protect against *M. tuberculosis* infection. The developed subunit vaccine could elicit innate and adaptive immune responses and protected against *Mtb* challenges and c-di-AMP being a mucosal adjuvant enhanced the innate immunity and is a preferred candidate for a mucosal vaccine against TB.

Since the speedy diagnosis of pulmonary tuberculosis (PTB) remains a task during clinical practice. Peng et al. addressed this issue by optimizing an algorithm for rapid diagnosis of PTB in a real-world setting. Their significant contribution by concurrently performing AFB smear and Xpert MTB/RIF assay on sputum and/or BALF could aid in rapid diagnosis of PTB and nontuberculous mycobacteria (NTM) infections in a real-world high-burden setting using reasonable sample replicates.

A few bacilli can hide and live inside the host mesenchymal stem cells (MSC) and that leads to futile therapeutics often. Aqdas et al. presented an exhilarating work to clear this cellular bacillus using immunotherapy-based approaches. Cumulative signaling through NOD-2 and TLR-4 could eliminate *M. tuberculosis* and significantly reduce the intracellular survival of *Mtb* in the MSC. Overall, their results suggest that the triggering through N2.T4 can be a future method of immunotherapy to eliminate the *Mtb* concealed inside the MSC.

STAT3 had a great effect on fast-acting innate immunity against *Mtb* and it also has an important role in biological balance. Wang et al. hypothesized that STAT3 SNP down-regulation of STAT3 leads to a change in susceptibility to TB in humans. They experimented with their hypothesis in a case-control study of TB patients and healthy control (HC) subjects, then conducted a functional analysis using cellular models. Their innovative finding suggests that low constitutive STAT3 derived from the T/A genotype/T-A haplotype acts to down-regulate STAT3, depressing multiple anti-mycobacterial pathways/mechanisms downstream, which leads to an enhanced mycobacterial infection or TB in high-risk individuals.

Jorgensen et al. explored the effect of cyclooxygenase 2 inhibitor (COX-2i) treatment on eicosanoid levels and signaling pathways in monocytes. Eicosanoids and intracellular signaling pathways are potential targets for host-directed therapy (HDT) in TB. The systematic outcome of this study showed that COX-2i may reduce excess inflammation in TB *via* the lipoxygenase (LOX) pathway in addition to modulation of phosphorylation patterns in monocytes. Immunomodulatory effects of adjunctive COX-2i in TB may be used as an HDT strategy.

Indeed, *Mtb* inhibits autophagy to support its survival in host cells, even though the molecular mechanisms behind this process are not well established. Sengupta et al. magnificently established the mechanistic way of the *Mtb* inhibition of autophagy. They identified the *Mtb* phosphoribosyltransferase (MTB-PRT) inhibits autophagy in an mTOR-independent manner in *Mtb* infected macrophages.

People with type 2 diabetes (T2D) are a known risk factor for TB. Therefore, T2D increases the individual’s susceptibility to incident TB. Sinha et al. attentively developed a preclinical model of pre-diabetes and TB. The developed murine model offers the opportunity to further study the underlying immunological, metabolic, and endocrine mechanisms of the association between T2D and TB. Their finding demonstrated that pre-diabetes increases susceptibility to TB, but a high body mass index without dysglycemia is protective.

Chen et al. systematically discovered novel potential diagnostic serum biomarkers of metabolomics in osteoarticular TB patients. Osteoarticular TB is one of the forms of extrapulmonary TB. As it is already described above that TB is caused by Mtb infection. Since metabolomics is used to study the changes in the body’s metabolites during different states, it is important means of discovery of disease-related metabolic biomarkers and the corresponding mechanism research. This group has identified several biomarkers and they had high diagnostic values.

Miliary pulmonary TB in pregnant women after *in vitro* fertilization-embryo transfer (IVF-ET) leads to poor outcomes, which needs more emphasis. Dong et al. analyzed the clinical features and risk factors in pregnant women with miliary pulmonary TB after IVF-ET. Their finding denotes that tube infertility with underscreened or untreated TB is a risk factor for miliary TB during pregnancy after IVF-ET.

This exceptional compilation of articles on our Research Topic gives new insights into the risk factors associated with *Mtb*, molecular pathogenesis, improvised diagnosis, and therapeutics. This also offers novel approaches to fight this notorious pathogen *Mtb*. We thank all the reviewers for their comments that improvised the manuscripts, and we also thank all the authors for their novel exceptional contributions.

## Author contributions

The author confirms being the sole contributor of this work and has approved it for publication.

## Conflict of interest

The author declares that the research was conducted in the absence of any commercial or financial relationships that could be construed as a potential conflict of interest.

## Publisher’s note

All claims expressed in this article are solely those of the authors and do not necessarily represent those of their affiliated organizations, or those of the publisher, the editors and the reviewers. Any product that may be evaluated in this article, or claim that may be made by its manufacturer, is not guaranteed or endorsed by the publisher.

